# Alleviating Environmental Health Disparities Through Community Science and Data Integration

**DOI:** 10.3389/fsufs.2021.620470

**Published:** 2021-06-10

**Authors:** Mónica D. Ramírez-Andreotta, Ramona Walls, Ken Youens-Clark, Kai Blumberg, Katherine E. Isaacs, Dorsey Kaufmann, Raina M. Maier

**Affiliations:** 1 Department of Environmental Science, University of Arizona, Tucson, AZ, United States; 2 Mel and Enid Zuckerman College of Public Health’s Division of Community, Environment and Policy, University of Arizona, Tucson, AZ, United States; 3 BIO5 Institute, University of Arizona, Tucson, AZ, United States; 4 Department of Biosystems Engineering, University of Arizona, Tucson, AZ, United States; 5 Department of Computer Science, University of Arizona, Tucson, AZ, United States

**Keywords:** citizen science, community science, interoperability, FAIR principles, environmental health, community resiliency

## Abstract

Environmental contamination is a fundamental determinant of health and well-being, and when the environment is compromised, vulnerabilities are generated. The complex challenges associated with environmental health and food security are influenced by current and emerging political, social, economic, and environmental contexts. To solve these “wicked” dilemmas, disparate public health surveillance efforts are conducted by local, state, and federal agencies. More recently, citizen/community science (CS) monitoring efforts are providing site-specific data. One of the biggest challenges in using these government datasets, let alone incorporating CS data, for a holistic assessment of environmental exposure is data management and interoperability. To facilitate a more holistic perspective and approach to solution generation, we have developed a method to provide a common data model that will allow environmental health researchers working at different scales and research domains to exchange data and ask new questions. We anticipate that this method will help to address environmental health disparities, which are unjust and avoidable, while ensuring CS datasets are ethically integrated to achieve environmental justice. Specifically, we used a transdisciplinary research framework to develop a methodology to integrate CS data with existing governmental environmental monitoring and social attribute data (vulnerability and resilience variables) that span across 10 different federal and state agencies. A key challenge in integrating such different datasets is the lack of widely adopted ontologies for vulnerability and resiliency factors. In addition to following the best practice of submitting new term requests to existing ontologies to fill gaps, we have also created an application ontology, the Superfund Research Project Data Interface Ontology (SRPDIO).

## INTRODUCTION

### Research Context

Pollution is now the leading global cause of premature death and disease (Landrigan et al., 2018). This crisis is currently being addressed through environmental monitoring and public health surveillance efforts that are conducted by local, state, and federal agencies. Most states have environmental quality and health departments, which have the major responsibility for environmental protection and the health and safety of the population. The U.S. federal government has a number of overarching environmental and public health agencies, including the Centers for Disease Control and Prevention, the National Institutes of Health (NIH), the Food and Drug Administration, the Environmental Protection Agency (USEPA), Geological Survey (USGS), and Department of Agriculture (USDA). In addition, two other entities can play important roles in environmental quality and public health; non-governmental organizations (NGOs) and universities, both public and private, that receive extramural funding to conduct environmental quality and public health research.

One of the challenges with these efforts is that the datasets generated by each group are independent and siloed from one another, leading to a lack of standardization, interoperability, application of FAIR (Findable, Accessible, Interoperable, and Reusable) principles of data management, and stewardship (Wilkinson et al., 2016). A second challenge is that community members are rarely involved in environmental monitoring projects. Professionally paid researchers are missing key opportunities to partner with vulnerable communities, collect high resolution data, and incorporate potential exposure routes that may otherwise be overlooked (e.g., Garcia et al., 2013; Ramirez-Andreotta et al., 2013a,b; Ramirez-Andreotta et al., 2014; Dhillon, 2017; Manjón et al., 2020).

Public Participation in Scientific Research (PPSR) efforts such as citizen and community science programs (referred to as CS hereafter) can be used to address the latter challenge. PPSR is broadly defined as partnerships between scientists and non-scientists in which authentic data are collected, shared, and analyzed (e.g., Shirk et al., 2012). Such efforts have dramatically increased in the past few years (Pocock et al., 2017), and it is anticipated that this approach will permanently change the face of how scientific data are collected and who collects it. Incorporation of CS into research efforts has exciting potential due to the vast amount of data and observations that can be collected by the general public. What is most remarkable about this methodology, is the potential to redistribute power, democratize science and achieve environmental justice (Ottinger, 2010; Pandya, 2012; Allen, 2018). CS efforts are increasingly being directed toward environmental monitoring and will be key and necessary to fully understanding the environmental determinants of chronic disease. Such monitoring information will provide the scientific basis for future prevention of environmental exposures and motivating action (Morello-Frosch et al., 2009).

The critical obstacle to using CS data in assessment of environmental exposure is data management and interoperability. As laid out in the 2016 report, “Stakeholder Analysis: International Citizen Science Stakeholder Analysis on Data Interoperability” there is empirical evidence for the importance of data standards in CS, most noteworthy is that some authorities may not use CS data because of “uncertainty about data quality assurance and quality control measures, and a lack of data standardization practices” (Gobel et al., 2016). Yet studies have confirmed that CS models can provide accurate and reliable data (e.g., Haklay, 2010; Gollan et al., 2012; Nagy et al., 2012; Tregidgo et al., 2013; Hecker et al., 2018). In order to move these data beyond disciplinary and stakeholder boundaries, data management and quality assurance is required (Haklay, 2017; Hecker et al., 2018), along with internal support and tools to effectively address the problems identified by CS. For example, the USEPA is supporting CS projects and has generated quality assurance guidance documents that include templates and handbooks to inform community members and other federal and state agencies (USEPA, 2020).

The scarcity of FAIR data (Wilkinson et al., 2016) in CS is not only unfortunate for the progression of science, but unethical. Thousands of people are contributing/participating to CS programs and co-generating datasets; dedicating their time and resources hoping that their efforts will create change and positive social-ecological outcomes (e.g., Shirk et al., 2012; Ramirez-Andreotta et al., 2015). The lack of data standardization and application of FAIR principles in government-, NGO-, university-, and CS-based efforts slows down the ability and efficiency to address environmental health disparities. Further, most public health environmental health monitoring efforts use an epidemiological approach, but it is known that epidemiology alone cannot adequately detect the effects of toxic exposures on human health (Brown, 1992; Brown and Mikkelsen, 1997). Specifically, a fundamental and critical challenge that exists in environmental justice communities is the need to account for interrelated effects of culturally-diverse and economically-disadvantaged groups with toxic exposures. Another challenge is accounting for community resiliency: the sustained ability of a community to withstand and recover from adversity (Plough et al., 2013). Community resiliency comprises the enduring capacity of geographically, politically, or affinity-bound communities to define and account for their vulnerabilities and develop capabilities to prevent, withstand, or mitigate (Abramson et al., 2015).

We hypothesize that FAIR principles can be applied to facilitate the seamless integration of CS/government/NGO/university datasets and allow the inclusion of both community vulnerabilities and resiliencies in the environmental health assessment process. This approach will allow incorporation of all viable data as well as scaling of datasets, a process that can be expected to increase the efficiency and impact of public health intervention efforts.

To address this hypothesis, we have developed a methodology to make environmental health CS data FAIR. This methodology: (1) integrates CS environmental monitoring data with other data sets to enhance discoverability and reuse of data for research translation and (2) enables better hypothesis generation. An anticipated result of this integration effort is that it will help determine if and how community-level resiliencies may combat environmental health vulnerabilities. In this methodology, we use ontologies to combine a CS dataset with existing governmental environmental monitoring and community resiliency data. An ontology is a forma specification of the concepts in a domain and the relationships among them (Gruber, 1993). The use of ontologies is a key component of FAIR data, because ontologies can transform free-text descriptions into structured, standardized machine-readable data, improving findability, interoperability, and reusability.

The CS dataset used in this research is Gardenroots, a co-created CS program. Gardenroots sees gardens as hubs for environmental health research and literacy with the goals of: engaging community members in the environmental monitoring and exposure science process; evaluating environmental quality (water, soil, and homegrown vegetables) and potential exposure routes; and designing personalized and community-based data sharing experiences to support environmental action and decision-making (Ramirez-Andreotta et al., 2013a,b; Ramirez-Andreotta et al., 2015; Sandhaus et al., 2019). CS programs such as Gardenroots demonstrate how community-engaged environmental monitoring efforts have informed local food gardening practices. By working together to determine soil quality and contaminant concentrations, Gardenroots helps sustain community and home gardens efforts while reducing chemical exposures. This is critical because community and home gardening efforts help address social and economic constraints on health by increasing access to wholesome foods, improving community building efforts, enhancing emotional well-being, creating green space, and reducing the cost of food (Ness and Powles, 1997; Armstrong, 2000; Teig et al., 2009; Ramirez-Andreotta et al., 2019). Gardenroots builds on individual- and community-level resiliencies and combats environmental health vulnerabilities, helping to ensure pollution does not interfere with local gardening efforts. However, Gardenroots is site-specific. We use this dataset to demonstrate the possibility of integration of these data with other state and federal datasets related to soil quality, food production, health, etc. This integration not only increases the spatial resolution and understanding of pollution, but also has the potential to increase environmental health decision-making capacity.

## MATERIALS AND EQUIPMENT

### About Gardenroots

**FIGURE 1 |:**
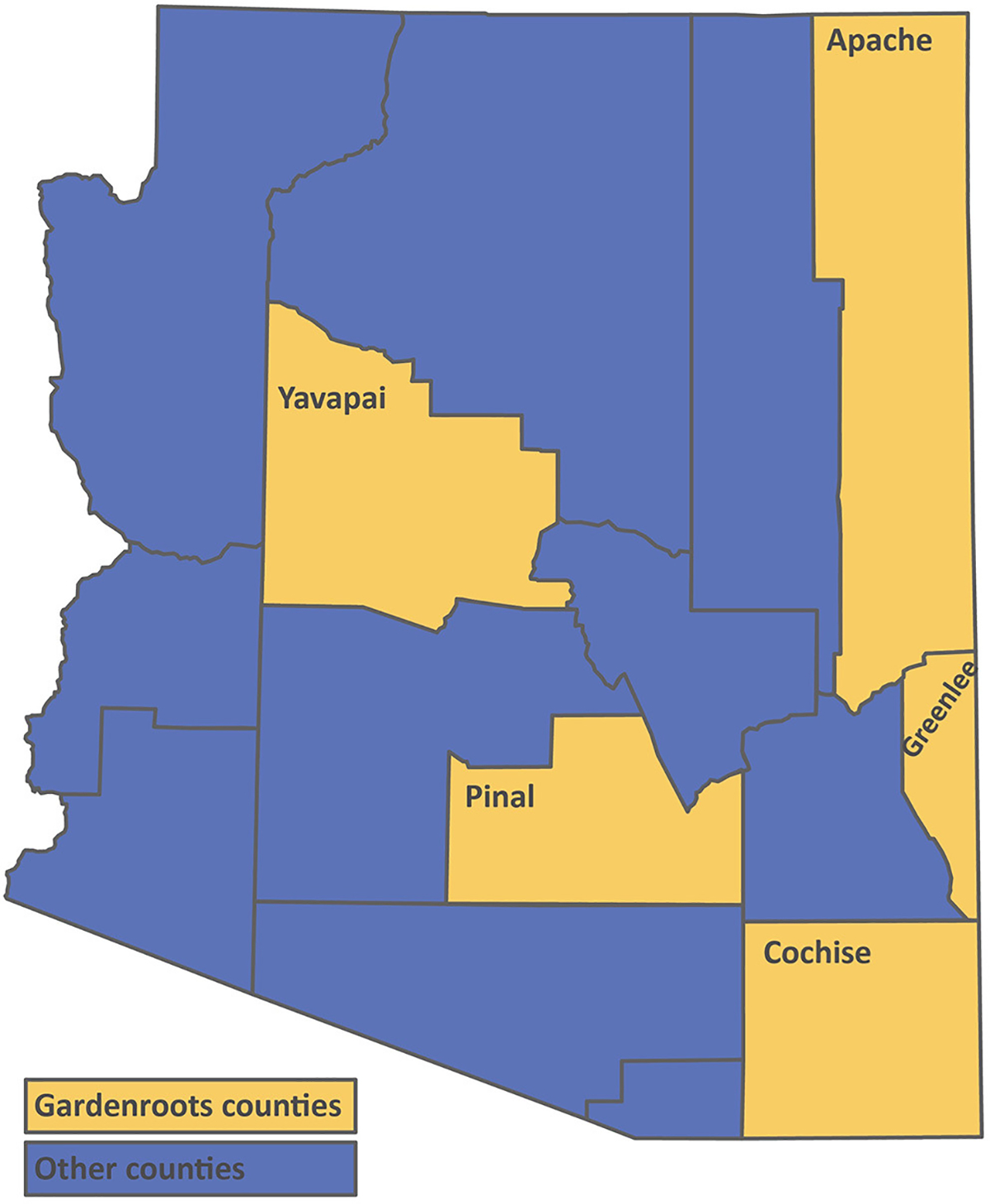
Map of participating Gardenroots communities in Arizona.

Gardenroots was established in 2010 in collaboration with a rural community neighboring a USEPA National Priorities List site under the Superfund program slated for cleanup due to uncontrolled hazardous waste (Ramirez-Andreotta et al., 2013a,b). The site comprises a large mine tailings pile that has been barren and subject to wind and water erosion since the 1960s as well as a closed smelter facility site. Both the mine tailings and the smelter site are contaminated with high levels of arsenic, lead, and zinc (USEPA, 2013). More recently, based on the results of a community needs assessment and ongoing community engagement in Arizona, Gardenroots was continued in summer 2015 and 2019 to help address additional community concerns regarding their soil, water, and/or plant quality. Since inception, Gardenroots has been implemented in nine communities nationwide and in AZ alone, more than 120 participants have been trained. Each Gardenroots participant completed a 2-h training on how to properly collect samples from a self-selected area. Community recruitment, trainings, and retention procedures have been previously described (Ramirez-Andreotta et al., 2015; Sandhaus et al., 2019). Typical locations included residential areas, community or school gardens, and local farms. Participants collected water, soil (yard and garden), and/or edible plant samples and submitted them to a centralized location for transport to the University of Arizona (UA). The dataset set reported here is from the following Arizona counties: Apache, Cochise, Greenlee, Pinal (Superior), and Yavapai (Dewey-Humboldt) (Figure 1). Each sample submitted and included in this dataset was analyzed for aluminum, arsenic, barium, beryllium, cadmium, chromium, copper, lead, manganese, nickel, and zinc concentrations in water (micrograms per liter, μg^−1^), soil (milligrams per kilogram, mg kg^−1^), and/or plant samples (mg kg^−1^). Field and laboratory methodologies have been previously described (Ramirez-Andreotta et al., 2013a,b; Manjón et al., 2020). All Gardenroots participants received their data (individual and aggregated) via visually-rich results booklets distributed at data sharing and community gathering events or by mail (Ramirez-Andreotta et al., 2015; Sandhaus et al., 2019). In this Methods paper, the Gardenroots CS data is being used as an example to generate a methodology for others to use and allow the seamless integration of other CS collected data with existing state and federal agency datasets.

### Data Management Materials and Equipment

We are using CyVerse (Merchant et al., 2016; https://cyverse.org/) as the primary data storage platform. CyVerse allows all project members to access and analyze data from a shared directory, thus reducing the risk of forking (having múltiple, divergent copies of the same dataset). Python code for data cleanup and processing are hosted on GitHub at https://github.com/UA-SRC-data/data_loaders. The combination of shared storage and public code allows us to track exactly what processing steps were carried out on each dataset and allows others to reproduce our results. Details on the usage of these platforms is included in section Integrating CS and Federal and State Data Sources.

## METHODS

### Federal and State Datasets

In addition to the Gardenroots CS dataset, data were pulled from existing state and federal programs (Tables 1, 2). These datasets were selected to provide a comprehensive understanding of the possible vulnerabilities and resiliencies in Arizona rural, with special attention on medically-underserved communities that neighbor resource extraction activities. With an understanding of the possible vulnerabilities and resiliencies, efforts will be placed on gathering and juxtaposing variables to see for example, where a community has a tremendous amount of resiliency that has not been tapped for sustainability, environmental quality, and/or justice purposes or vice versa, where an area is suffering and community capacity efforts are in need.

### Vulnerability Datasets

To determine vulnerability (function of the exposure and sensitivity of system, e.g., Cutter, 1996; Adger and Neil Adger, 2006; Cutter et al., 2008), datasets selected include (Table 1):

- Quality of Environment
- Quality of Health
- Social Attributes
- Access to Food.

### Community Resilience Datasets

Abramson et al. (2015) proposed the Resilience Activation Framework, a conceptual model of how access to social resources promote adaptation and rapid recovery within individuals and communities. This framework rests on six described principles and assumes that access to social services can activate resilience characteristics that are inherent in both individuals and communities, and that once activated, lead to better mental and physical health and well-being (Abramson et al., 2015). Using this proposed design, community resiliencies were collected from diverse datasets and are divided here into (Table 2):

- Economic Capital
- Human Capital
- Social Capital
- Political Capital.

### Integrating CS and Federal and State Data Sources

#### Data Processing SOP

To maximize the FAIRness of the data collected and analyzed as part of this project, we established a standard operating procedure (SOP) for all datasets that stores raw and processed data in shared folders, tracks all data processing steps, standardizes variables to existing ontologies wherever possible, and publishes standardized data to trusted repositories. Individual technologies in this SOP could be replaced with others of similar functionality. The full SOP is available at https://github.com/UA-SRC-data/data_loaders/blob/master/README.md, but in brief it describes how to:

1. Ensure a copy of the raw data is preserved and sufficiently documented: Gather raw data and store data in a shared CyVerse folder under use case name, under “raw-data.” Include a readme file to readme in each raw folder with the link to the data source and a data dictionary defining variables if needed. Also document data sources in the readme file in the appropriate directory in the data_loaders code repository. Documenting each step of data analysis, including raw data, is crucial for reproducibility.
2. Convert all data to a common format so that it can be integrated: Preprocess data to convert to CSV files with a single sheet per file and a single header row. Standardize column headers by mapping to ontology templates. Output as a CSV file and store on CyVerse under “pre-processed.” Data processing scripts are available on a per dataset basis at https://github.com/UA-SRC-data/data_loaders
3. Visualize and validate data: Loading data into the project MySQL or other database allows for preliminary visualization, the first step in most big data projects. This acts as a validation step that allows us to identify outliers and errors in the data such as incorrect units, mapping errors during step 2, or incorrect datatypes.
4. Run data through the Ontology Data Pipeline (https://github.com/biocodellc/ontology-data-pipeline) to convert to graph format. In addition to standardizing the data, the ontology can infer new facts such as hierarchical classification, which enhances searching. More details on the use of ontologies is included in section Standardizing Vulnerability and Resiliency Variables to Ontologies.
5. Output final datasets including standardized versions of datasets for publication as well as complete versions of dataset to use in the visualization portal.

#### Decision-Making and Standardization Practices

Integrating data from multiple databases requires many decisions regarding which data to include, how to carry forward missing or other special values, and how to harmonize data collected at different spatial scales or time points. It is critical to document these decisions and ensure that documentation accompanies any published datasets.

##### Managing Non-numerical Values in Numerical Fields

It is common for data sources to include non-numerical values in certain cells where a numerical value is expected. Data from the National Water Quality Monitoring Council includes values like “<0.02” to indicate metal(loid) concentrations below the limit of detection (LOD). Such values will not pass validation and cannot be computed on, so researchers must decide how to use them, which is challenging for data that were collected by a third party. In the Gardenroots dataset, all values below LOD are recorded as LOD/√ 2 so that they can be included in analyses (USEPA, 1991; Helsel, 2011). Because our database (step 3 in section Data processing SOP) specifies datatypes (e.g., float, string) for each field, it will automatically find values of this type that need to be addressed.

##### Variation in Spatial Granularity

Spatial resolution varies among datasets, including both point locations and shape files at the census block, block group, and tract level or county level. We chose the census block group as our preferred spatial resolution because it strikes a good balance between specificity and availability among different data sources, and because it is the resolution of Gardenroots data (see section below on privacy). Furthermore, for some datasets, such as the USEPA’s Environmental Justice Screening tool (EJSCREEN), limited data availability at finer resolution can lead to unacceptable levels of uncertainty (USEPA OECA, 2014). Some datasets are only available at the county level (e.g., USDA data), so any analyses at finer scales must include the uncertainty that comes from using county level data. Data at the point level (e.g., USGS water monitoring data) can be converted to block group using standard code libraries (see https://github.com/UA-SRC-data/data_loaders/tree/master/point2shape), with the recognition that this introduces uncertainty for that block group. Therefore, processed datasets must include annotations that data were converted from point to shape file. In addition to the spatial resolutions listed above, we are also adding spatial files for different boundary types, to represent, for example, tribal homelands and Primary Care Areas.

##### Variation in Temporal Granularity

The time of data collection also varied among datasets. Gardenroots data were collected over multiple years (2010–11, 2015–16, 2019), sometimes with multiple data points for the same location. Some federal datasets are available for multiple years, while others are available for a single year only. For those that are available over multiple years (e.g., EJSCREEN), we chose to use only data from the most recent year. Because our integration uses only datasets from the period of 2010–2020, we make the assumption that they are comparable, but variation in year collected introduces additional uncertainty. Often, when data at a broader temporal resolution are combined with date where an exact date is known, a specific date will be assigned. For example, a data point for 2018 might be assigned a date of January 1, 2018, to be compatible with other data points for which the day of the year is known. This introduces a false sense of precision. When integrating data at different scales (spatial or temporal), the integration must usually happen at the largest scale, even if this means losing information from more precise dataset.

#### Protecting Privacy of CS Data

When working with CS data such as Gardenroots, it is critical to preserve the privacy of participants. Before data collection, all participants were consented under the University of Arizona Institutional Review Board as an approved project for learning research. Although the UA currently does not see environmental monitoring as a “type” of human research, the name, location, and reported-back environmental monitoring data were deidentified to preserve participant privacy. It is clear that there is an ethical duty to report data back to participants, but once the participant has that data, are there ethical or legal implications? Do they have to disclose when selling their home? Renting? Having family members visit who are considered a member of the sensitive population (i.e., under five, over 65, and/or have a preexisting condition)? Goho (2016) reviewed and explored the potential legal duties of study participants once they have participated in a residential exposure study and have received their personalized data results. It was concluded that there are both ethical and legal implications that researchers and community researchers need to consider, highlighting how data privacy and preservation is critical to CS data science efforts. Based on the above and previous efforts, a solution was reached where community data reported herein was kept to the geographic resolution of the census block when the census block includes at least 10 residences and the census block group otherwise.

#### Standardizing Vulnerability and Resiliency Variables to Ontologies

Ontologies are standardized terminologies that provide logical (understood by computers) and text (understood by humans) definitions to reduce ambiguity about the meaning of data. A key challenge in integrating such disparate datasets (with variables ranging from metal(loid) concentration in garden soil to median household income to proximity to grocery stores) is the lack of widely adopted ontologies for vulnerability and resiliency factors. Environmental vulnerability terms for chemical exposures have the best existing coverage in ontologies, due to chemical terms in Chemical Entities of Biological Interest ontology (CheBI, de Matos et al., 2009) and environmental quality terms in the Environment Ontology (ENVO, Buttigieg et al., 2013, 2016). We follow the best practice of submitting new term requests to existing ontologies to fill gaps, but that process can be slow. Therefore, we have created an application ontology, the University of Arizona Superfund Research Project Data Interface Ontology (SRPDIO, https://github.com/UA-SRC-data/srpdio) to meet our pressing data integration needs. Superfund Research Project Data Interface Ontology reuses terms from the ENVO, CheBI, the Exposure Ontology (Mattingly et al., 2012), and other ontologies to standardize variable names across datasets. We are working with ENVO curators to move physio-chemical parameters such as metal(loid) concentration or electroconductivity into ENVO, where they can be more broadly reused. For variables that have no ontology (e.g., number of registered voters or proximity to EPA Risk Management Plan Facilities), we are creating terms within the SRPDIO to explicitly define each variable. We plan to work with the larger environmental health community to develop ontologies around social vulnerability and resiliency factors in the future.

## RESULTS

### Integrated Datasets That Permit New Environmental Health Studies

The Gardenroots CS data were integrated with existing governmental environmental monitoring data to create a more holistic story that includes vulnerability and resiliency data from these rural, medically-underserved communities. We integrated typically siloed/separated datasets including datasets that are intentionally segregated based on who collected the data. The integration of these datasets allowed for the generation of the proposed questions in Table 3 that we anticipate answering (see Figures 3–5 for examples). The vulnerability and resiliency data in Tables 1, 2 are in various stages of processing with the SOP described in section Data processing SOP. Metal(loid) concentration data from Gardenroots, National Water Quality Monitoring Council, and USGS; pollution-related data from US EPA’s EJSCREEN; and social data from the U.S. Census Bureau’s American Community Survey (ACS) have been preprocessed, and validated using our internal database (step 3 in section Data processing SOP). These datasets are available in an archived release of our GitHub repository (Youens-Clark et al., 2020) in files named “scrutinizer.csv” under their corresponding directories, along with the code that generated them. Because Gardenroots contains multiple datasets, the pre-processed data are instead in a directory named “scrutinizer” with separate files for plant and soil data. Food access data from USDA’s Economic Research Service, health data from the National Center for Health Statistics, and NIH’s Health Resources and Service Administration’s Health Professional Shortage Area data have been downloaded and stored in our shared CyVerse directory, but still require standardization. We do not publish those datasets, as they are available from the original sources, listed in Tables 1, 2. To access data from the Center for Disease Control’s Behavioral Risk Factor Surveillance System (BRFSS), we will need to submit a request and complete an application. CDC restricts re-publication of BRFSS data, so although we plan to use them in our integrated data modeling work, we will not be able to publish them. Recognizing the need for privacy, FAIR principles do not require that data be open, but they do require adequate description. Therefore, we will provide full metadata for any BRFSS data we use. Arizona voter data, available from https://azsos.gov/precinct-level-results-county-2018-general-election, will require additional processing for some counties to extract the desired variables, because county- level data are not reported consistently.

Though we have not yet used this methodology extensively, Figures 3–5 are example visualizations generated from the integration of selective datasets at the county level, demonstrating initial and further anticipated results. We recognize that causality cannot be inferred, but these examples show how the database can help inform hypothesis generation and identify counties that are suffering more from selected health outcomes and/or environmental quality challenges. These visualizations were created to align with the questions posed in Table 3 to illustrate the breadth of this methodology. For example, arsenic and chromium (inhalation route only) are recognized as human carcinogens by USEPA, while cadmium and lead are classified as probable carcinogens (e.g. USEPA Integrated Risk Information System, USEPA, 2021). Figure 3 supports hypothesis generation, specifically asking whether arsenic, cadmium, chromium, and/or lead soil concentrations occur in counties with high incidence rates of the most commonly observed cancer types, informing questions 3–4 in Table 3. We see that Mohave county experiences bladder, lung, kidney and pancreatic cancers, but is only impacted by chromium in soil, whereas Yavapai county is impacted by all metal(loid)s except cadmium and the bladder and lung cancer incidence rates are among the top five. Greenlee county has the highest concentrations of cadmium, chromium, and copper (currently not classified as a human carcinogen), however the Arizona Department of Health Services dataset is missing selected cancer incidence rates, which we will gather from another source listed in Table 1.

In addition to cancer, studies have also observed that arsenic exposure is associated with an increased risk of developing a number of diseases, including cardiovascular disease and type II diabetes (Sears and Genuis, 2012; Naujokas et al., 2013). Currently, University of Arizona Superfund researchers are working to determine how chronic exposure to mine wastes that contain arsenic contributes to the development of diabetes. Figure 4 examines the prevalence of diagnosed diabetes and obesity along with major mining activities in Arizona (Niemuth, 2015; Centers for Disease Control and Prevention, 2016; Richardson et al., 2019). Mining and industrial processes are primary sources of arsenic and heavy metal contamination in soil (Lee et al., 2005). Greenlee, Gila, Pinal, Navajo, Graham, La Paz, and Mohave populations have an incidence rate of diabetes and obesity at the medium level, 9–13.9 and 29.1—36.0%, respectively, as well as at least one major mine, informing questions 3—5 in Table 3.

Figure 5 highlights a human capital form of resiliency—the percent of internet subscriptions (dial-up and broadband, cellular data plan, and satellite internet services) in Arizona counties. Internet service is a form of resiliency, indicating potential technical literacy and access to information. The highest percentage of internet service is 35.88% in Yavapai county, followed by Pima, Mohave, and Maricopa counties. This information indicates that researchers, government agencies, and other organizations cannot solely rely on websites for information dissemination, informing question 11 in Table 3.

### New Ontology Terms

#### Metal(loid) Environmental Monitoring Data

**FIGURE 2 |:**
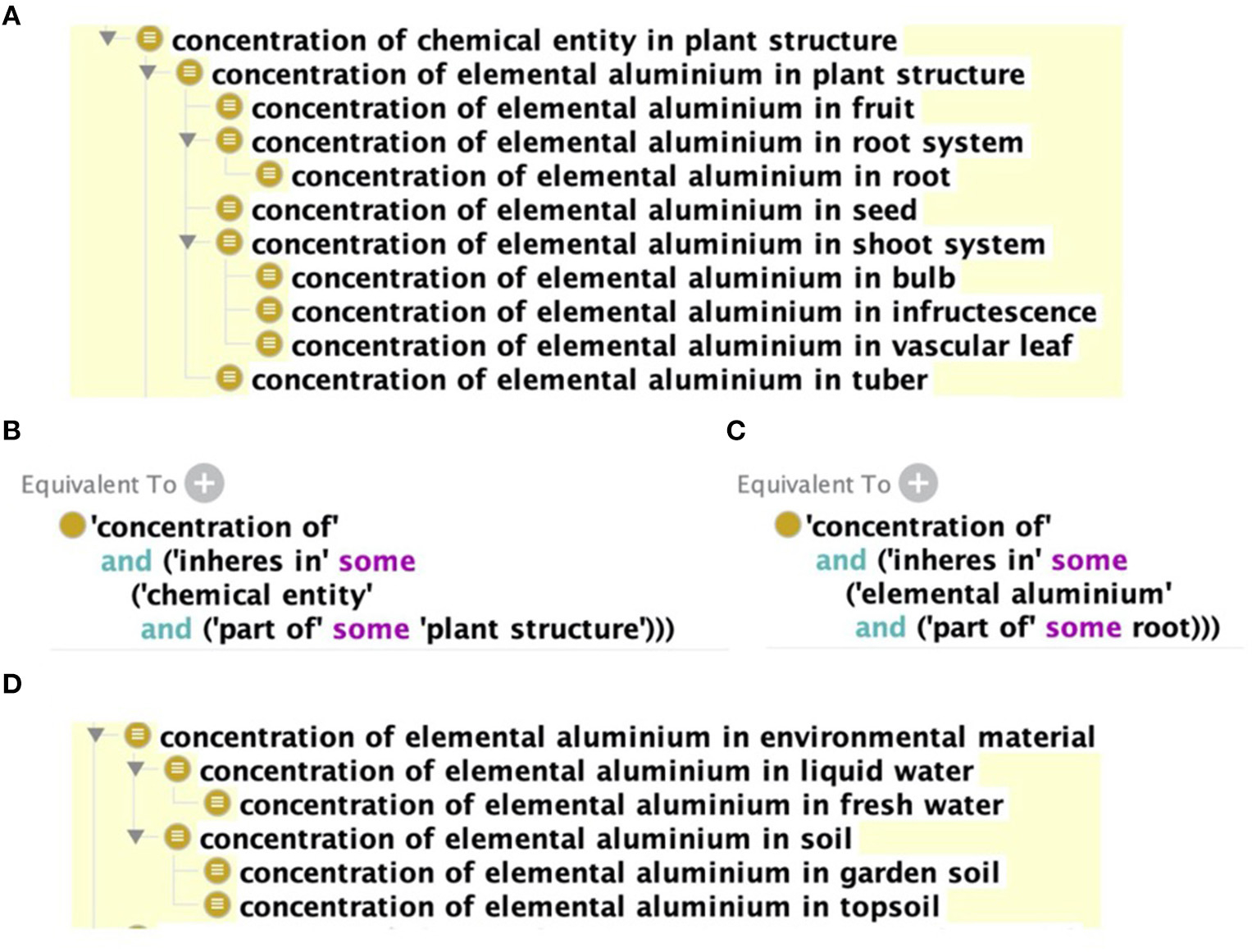
**(A)** ‘ “he hierarchy of terms for the “concentration of aluminium in plant structure.” Plant structure terms are imported from the Plant Ontology (Cooper et al., 2013) and chemical terms are imported from ChEBI. **(B)** The logical definition of “concentration of chemical entities in plant structure.” **(C)** The logical definition of “concentration of elemental aluminium in root.” An ontological reasoner uses these logical definitions to infer the hierarchy shown in **(A)**. **(D)** The hierarchy for “concentration of elemental aluminium in environmental material” is generated similarly to the hierarchy for concentrations in plant structures. Note that ChEBI is an international ontology that uses the British spelling “aluminium” shown in the figure, but our search engine includes the American spelling “aluminum”.

The initial draft of the SRPDIO and the code used to generate it are available at https://github.com/UA-SRC-data/srpdio, with the first official release in November 2020. A key component of the SRPDIO is the creation of new ontology terms for concentrations of metal(loid)s in environmental materials and plant structures. We use logical definitions for these terms that allow the ontological reasoner to automatically build complex hierarchies of metal(loid) concentrations (Figure 2). The logical definitions (Figures 2B,C) follow an ontology design pattern established in ENVO and the Plant Trait Ontology (TO, Cooper et al., 2018) to define terms for concentrations. “Inheres in” comes from the widely used Relations Ontology (Wg, 2020). It is used to relate a quality (in this case, “concentration of”) and the entity that has that quality (in this case a “plant structure” or a “material entity”). The ontological hierarchies support advanced queries, such as “find all data on any metal in a plant structure” or “find all data on zinc contamination in any material.” These terms and definitions were created in the SRPDIO but will be moved to the Environment Ontology with an upcoming ENVO release.

#### Sociodemographic Data

Another key component of the SRPDIO is the development of new ontology terms for sociodemographic variables. Currently, there is not a fully developed ontology for sociodemographic data, such as the information collected in the U.S. Census Bureau’s ACS. This was acknowledged as a main concern in Gobel et al. (2016), where interviewed stakeholders reported that current interoperability efforts are biased and limited to the natural sciences. Interviewees were critical that social science standards were absent from discussions, highlighting that any proposed interface and standardization effort would need to be accessible to a wide range of projects and research methodologies. Here, we acknowledge this bias and that the data science efforts have traditionally focused on the natural sciences, entailing observational data, and are not applicable to all forms of knowledge (Gobel et al., 2016). Another issue highlighted by interviewees in the aforementioned Stakeholder Analysis, was the lack of clarity on how to treat data gathered on participants including sociodemographic information and participant evaluations. As highlighted in section Protecting privacy of CS data, we have proposed a solution where community data can be reported while protecting privacy.

## DISCUSSION

### Solving Environmental Health Challenges With Transdisciplinary Data Science

This data science methods paper demonstrates the integrated framework needed to solve the challenges of interoperability within the environmental health sciences as well as how to integrate CS data. We have developed a methodology to make environmental health CS data FAIR, while also integrating other types of environmental health and social data to enhance discoverability, reuse of data for research translation, and enable hypothesis generation. We are, to the best to our knowledge, among the first to develop ontology terms for:contaminant concentrations in various environmental media, and sociodemographic data. This effort is advancing the field, while also demonstrating how the designed data management system can be applied to other research questions and scenarios. An anticipated result of this integration effort is that it will help the field determine if and how community-level resiliencies may combat environmental health vulnerabilities.

The complex challenges associated with environmental health and food security are influenced by current and emerging political, social, economic, and environmental contexts. To solve these “wicked” dilemmas (Rittel and Webber, 1973), we need methods to harness the public’s participation in research, conceptualize solutions, and strategize implementations at all levels of the ecological model of health to effectively design interventions (Bronfenbrenner, 1979; Richard et al., 2011). These challenges do not respect disciplinary boundaries. Therefore, transdisciplinary research efforts are needed *(e.g.,* Ramirez-Andreotta et al., 2014; Anderson et al., 2015; Pohl et al., 2017) that follow FAIR principles so that the varying knowledge sources can be interwoven (Anderson et al., 2015; Pohl et al., 2017). Based on the datasets highlighted and integrated in our case study, we do not necessarily need more data, we need integrated data management practices to solve the challenges of interoperability of CS data within the environmental health sciences.

### Place-Based Strategies to Mobilize Resiliencies

The data science methods reported here go beyond simply integrating CS environmental vulnerabilities datasets. Citizen and community science efforts can be viewed as place-based strategies to address public health challenges such as health promotion and environmental exposures. To build upon place-based strategies and social processes (e.g., Ness and Powles, 1997; Armstrong, 2000; Teig et al., 2009), we combined CS data with data on the communities’ human, social, and political capital to help inform how rural mining populations can mitigate potential chronic exposures and rebound when their ecosystem has been negatively impacted. For example, to combat natural disasters, Bergstrand et al. (2015) mapped social vulnerability and community resilience to visualize community risks as well as their capacities for recovery and adaptation.

**FIGURE 3 |:**
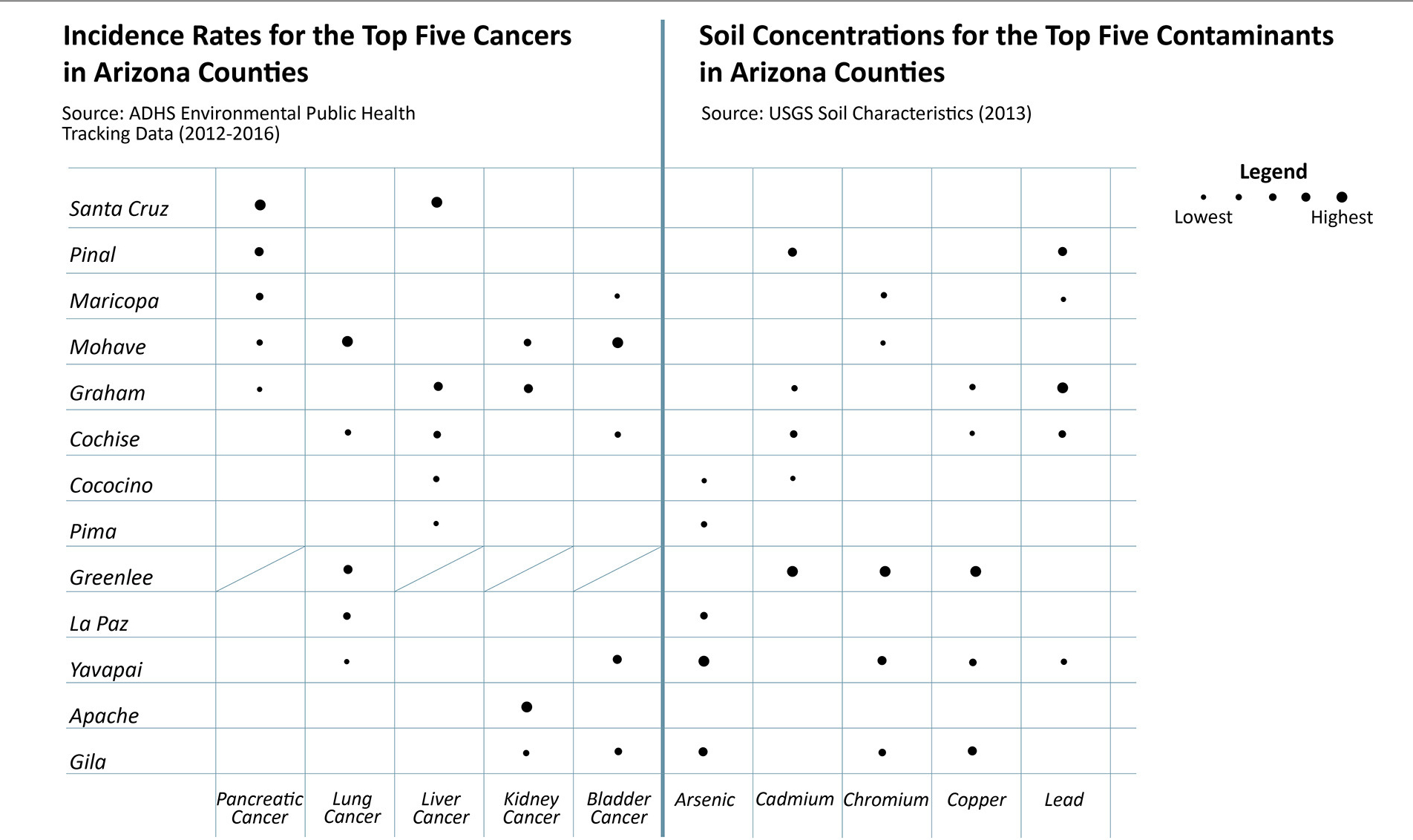
A visuazation generated from selective datasets to qualitatively describe cáncer incidence rates and soil contaminant concentrations.

**FIGURE 4 |:**
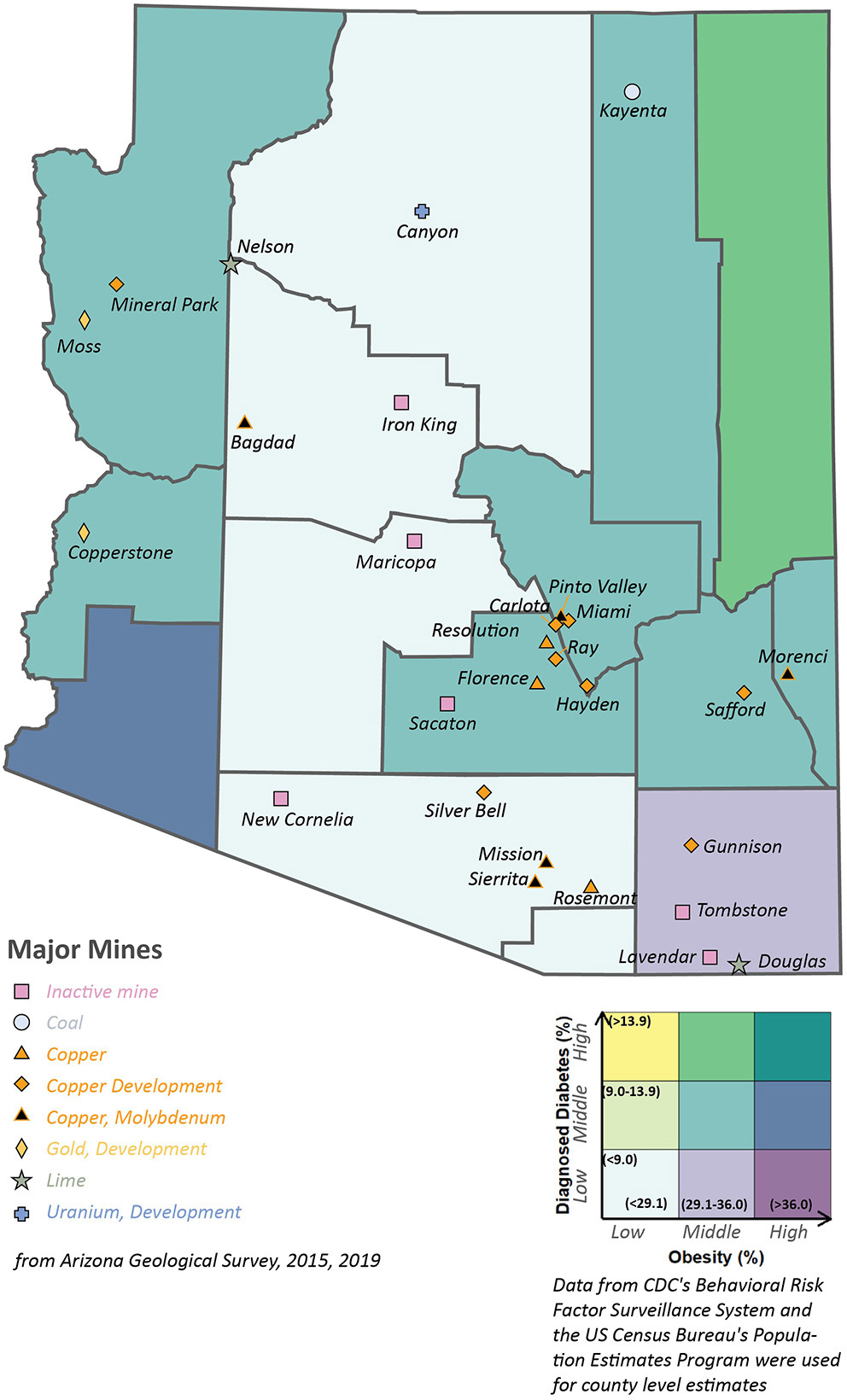
Major mines and prevalence of diagnosed diabetes and obesity in the state of Arizona.

**FIGURE 5 |:**
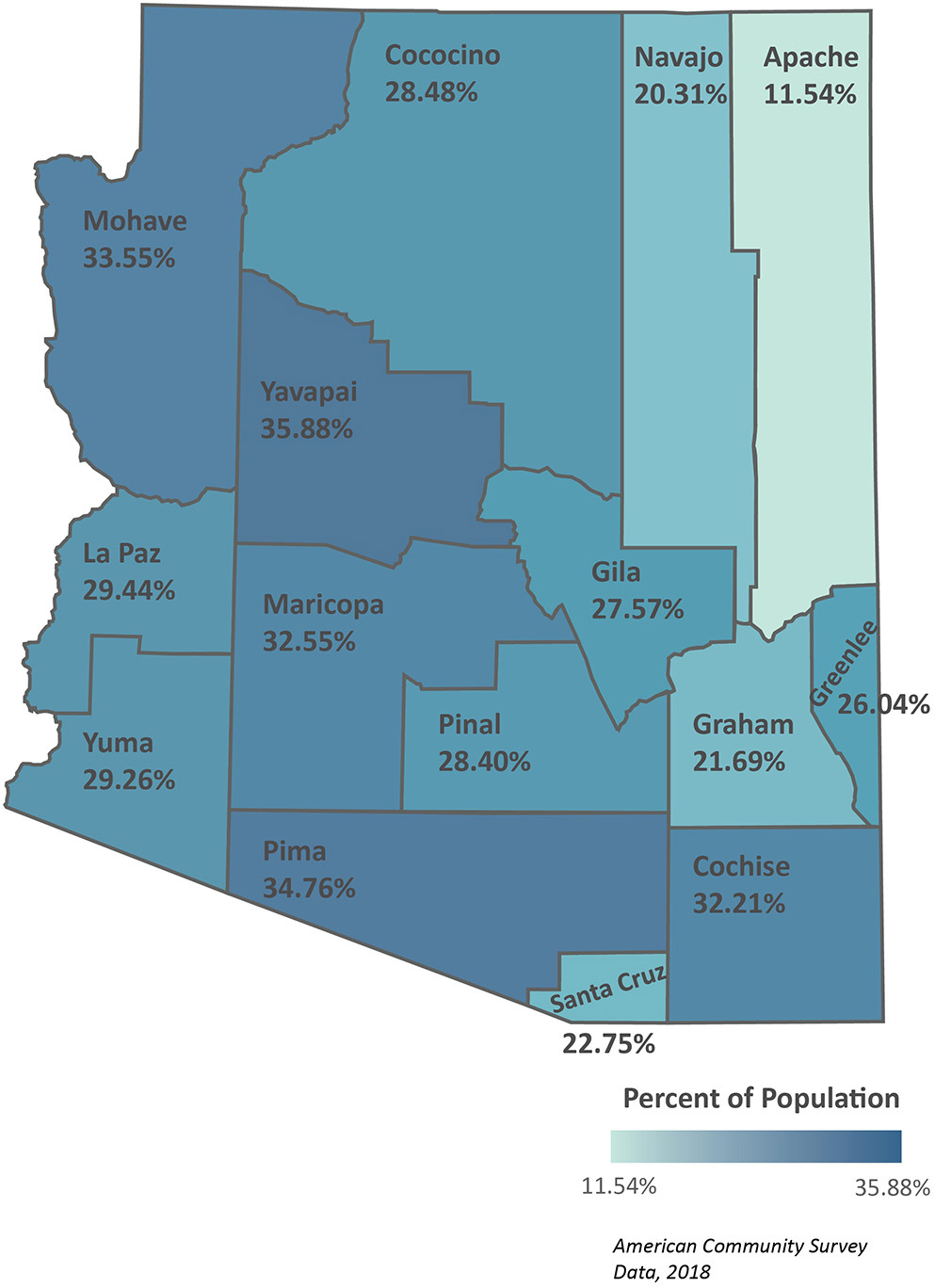
The percent of county populations with internet access

Regarding enabling hypothesis generation, we anticipate that the integrated Garderoots and government datasets will reveal new forms of community resiliency that can be mobilized to support and protect ecosystem services. Community resilience theory has become a key component of national policies across federal agencies because it provides a framework that embraces principles of equity and justice with a focus on building the capacities of populations both to mitigate disasters and to successfully rebound (Norris et al., 2008; Plough et al., 2013). Our methodology builds on this theory and can ultimately help to directly inform decision-making in these communities and identify critical areas for further study (Figures 3–5). Further, we anticipate that an understanding of soil quality from combining Gardenroots and USGS datasets will support provisioning services and inform where local food production efforts should be invested, addressing food deserts that have been highlighted in the USDA data. Alternatively, if local soils are not suited for crops, affected families can be connected to the Supplemental Nutrition Assistance and/or the Woman’s Infant and Children Programs for nutritional assistance. As a second example, in one community we had monthly meetings dedicated to identifying local concerns and priorities. A discussion regarding the need for occupational diversity has been initiated. The community does not want to be solely dependent on a local copper mine for economic prosperity as they recognize that copper ore is a finite resource. Thus, the community is interested in diversifying the types of jobs available in their community. Understanding current employment rates and labor force status, educational attainment, and the presence of a computing device and internet service/subscription at home, in the context of community educational, recreation and tourism, aesthetic, and cultural heritage values, can illuminate the best investments to make in social and human capital to facilitate occupational and economic diversification while protecting cultural resources.

### Determining Community-Level Resiliencies and How They May Combat Environmental Health Vulnerabilities

The resiliency literature has demonstrated that individual, family, social, and environment resources are critical for the successful recovery of a community or cultural system. Resiliency allows a given community to absorb a disturbance. This includes the ability to reorganize to meet the challenges of a change while still retaining the elements that make a community distinct (e.g., Healy, 2006; Fleming and Ledogar, 2008). Unfortunately, past efforts to understand resilience have focused on ecological systems and include socio-ecological systems to a much lesser extent (Bhamra et al., 2011). We anticipate that the lack of consideration of socio-economic systems is due to the absence of available information at different scales and research domains. But we argue that sustained community resiliency heavily relies on the improvement of social factors and this is a missed opportunity. Among these social factors are education, employment, and population well-being (Abramson et al., 2015).

The most important single predictor of health is socioeconomic status (e.g., Singh-Manoux et al., 2018; Kivimäki et al., 2020). Thus, one cannot separate socioeconomic status from environmental health vulnerabilities. However, efforts to improve environmental health need to include a better understanding and mobilization of current community-level resiliencies to help improve the socioeconomic status of the community as a whole. Table 3 illustrates the type of questions related to vulnerability and resiliency that our proposed framework and methodology would enable exploring. The anticipated outcome is illumination of improved and placed-based solutions to environmental health vulnerabilities (see section Place-Based Strategies to Mobilize Resiliencies).

### Power and Challenges of Interoperability

We have developed a method to provide a common data model that allows environmental health researchers working at different scales and research domains to exchange data. This method provides the ability to usefully incorporate such data, scaling the impact of any single dataset, be it from a single government, NGO, university, or CS source. We are currently working on an end-user/stakeholder analysis to determine “what works,” “what is missing,” and how to create the interactive data visualization approach that can be used for exploratory analysis and dissemination.

Key tasks for this goal include (Sedlmair et al., 2012): (1) Observations of current end-user/stakeholder’s analytical workflow and data visualization practices to prepare a validated visualization system; (2) Formative evaluation and usability studies of the validated visualization system with new end-users/stakeholders to ensure the visualizations meet stakeholder’s needs and answer their research questions; and (3) Development of a user-friendly web application that will support efforts to streamline data access, visualization, and analyses. In February 2021, we received University of Arizona Institutional Review Board Approval to start this analysis with local, state, federal, and community stakeholders. The newknowledge gained will aid in the creation of similar tools and workflows for use in other scientific contexts.

Modeling population- and factor-wide environmental effects using existing datasets from academia and federal agencies currently faces a number of challenges, including a limited number of samples in environmental datasets, which may prevent researchers from obtaining robust statistical confidence. Our method, which combines multiple data sources, helps to overcome the lack of power in an individual dataset by increasing the number of datasets available. Another key challenge in integrating CS data with public data and making it FAIR is the lack of existing standards and ontologies for environmental health data. The Children’s Health Exposure Analysis Resource ontology (Balshaw et al., 2017) provides broad coverage of environmental health indicators but lacks coverage of many important vulnerability and resiliency terms. We encourage environmental health researchers, especially those with knowledge of social and economic factors (which have the poorest ontological coverage) to get involved in community ontology development in order to support future data standardization and integration efforts. Our future work includes contributions to community ontologies such as ENVO and refinement of the SRPDIO.

## CONCLUSION

This effort has allowed for the development of a transdisciplinary data management (and eventually visualization) tool that we anticipate, will: (1) Help mitigate the human impacts of exposure to environmental contamination through effective research translation and community engagement driven by stakeholder-engaged research, and (2) Serve as a global resource for human and environmental health issues associated with contamination whether it is from a legacy site (as described in the Gardenroots example) or from a new or ongoing data source. It is expected that the interoperability efforts discussed herein, combined with the future end-user/stakeholder informed and validated data visualizations, will yield new insights into the factors that affect environmental health—both positively and negatively in communities.

## Figures and Tables

**TABLE 1 |:** Vulnerability datasets.

Data description	Dataset source	Year	Variable categories (*n* = number of variables in the category)
Social attributes	USEPA’s EJSCREEN	2019	Linguistically isolated (*n* = 3)
			Low income (*n* = 3)
			Minority population (*n* = 3)
			Less than highschool education (*n* = 3)
			Under age 5 (*n* = 3)
			Over age 64 (*n* = 3)
	American Community Survey	2019	Poverty status (*n* = 11)
Quality of environment	*Gardenroots* Data	2011–2019	Concentrations of metal(loid)s in water (*n* = 11)
			Concentrations of metal(loid)s in soil (*n* = 11)
			Concentrations of metal(loid)s in plants (*n* = 11)
			Concentrations of metal(loid)s in dust (*n* = 11)
	USEPA’s EJSCREEN	2019	Proximity to sources of pollution (*n* = 30)
			Air pollution (*n* = 21)
			Ozone level in air (*n* = 3)
			PM 2.5 in air (*n* = 3)
			Lead paint indicator (*n* = 3)
	National Water Quality Monitoring Council	2018	Water quality (*n* = 22)
	U.S. Geological Survey	2013	Soil characteristics (*n* = 42)
	Arizona Department of Health Services (ADHS) Environmental Health Public Tracking	2019 2019	Arsenic in community water systems (*n* = 1) Proximity of population and schools to highways
Quality of health	Behavioral Risk Factor Surveillance	2018	Diabetes (*n* = 1)
			Cancer (*n* = 2)
			Asthma (*n* = 2)
	ADHS Environmental Health Public Tracking	2016	Incidence of cancer (*n* = 14)
		2019	Hospitalizations for asthma (*n* = 3) Emergency department visits for asthma (*n* = 3)
	National Center for Health Statistics	2020	Prevalence of obesity or severe obesity among adults (*n* = 1)
Access to food	U.S. Department of Agriculture’s Economic Research Service (USDA ERS)	2020	State food insecurity (*n* = 6)

The most recent data available was used, with the exception of Gardenroots data, that spans from 2011 to 2020. Since the majority of the datasets had a large amount of measured variables, variables are grouped in categories in this table. The n value represents the number of variables in each category.

**TABLE 2 |:** Resiliency datasets.

Data description	Dataset source	Year	Variable categories (*n* = number of variables in the category)
Economic capital	American Community Survey	2019	Mortgage (*n* = 3) Employment (*n* = 782)
			Labor force status (*n* = 279)
Human capital	American Community Survey	2019	Education attainment (*n* = 27)
			Healthcare coverage (*n* = 25)
			Presence of computing device (*n* = 11)
			Internet service/subscription (*n* = 57)
	USDA ERS	2020	Access to women’s infants and children program (*n* = 15)
			Access to supplemental nutrition assistance program (*n* = 20)
Political capital	Arizona Secretary of State	2018	Registered voters (*n* = 1)
			Ballots casted (*n* = 1)
			Access to polling places (*n* = 1)
Social capital	Gardenroots Data	2013–2019	Number of *Arizona* communities participating in *Gardenroots* (*n* = 5)
	USDA ERS	2020	Proximity to grocery stores (*n* = 41)
			Store availability (*n* = 35)
			Food assistance (*n* = 59)
			Local foods (*n* = 96)
			Restaurants (*n* = 15)
	Human Resources and Service Administration-Health Professional Shortage Area	2019	Federally qualified health center (*n* = 1) Indian, tribal, and urban Indian organizations (*n* = 1) State mental hospital (*n* = 1)
			Rural health clinic (*n* = 1)
	ADHS Environmental Health Public Tracking	2019	Access to parks and elementary schools (*n* = 1)
		2013	Land use (*n* = 2)

The most recent data available was used, with the exception of Gardenroots data that spans from 2011 to 2020. Since the majority of the datasets had a large number of measured variables, the variables are grouped in categories. The n value represents the number of variables in each category.

**TABLE 3 |:** Questions to ask of the vulnerability and resiliency dataset to achieve environmental justice in communities neighboring active and legacy mining activities.

Questions	Dataset used
1. What is/are the major:	All datasets in Tables 1, 2
a. Vulnerability(ies)	
b. Resiliency(ies)	
2. Are we (all stakeholders) addressing them?	N/A
a. If not, how can we?	
3. Are mining communities disproportionately exposed to Arsenic? a. If so, what is/are the major arsenic contributor(s) to daily dose of arsenic?	• *Gardenroots Data* • USEPA’s EJSCREEN • National Water Quality Monitoring Counci • U.S. Geological Survey
4. Are mining communities suffering/experiencing cancer/diabetes/obesity/asthma disproportionately? a. Why? b. Is it due to rural health disparities? c. Access to nutritional foods and public health programming?	• ADHS Environmental Health Public Tracking • Behavioral Risk Factor Surveillance • National Health and Nutrition Examination Study • USDA ERS
5. Are mining communities with elevated arsenic concentrations suffering/experiencing cancer/diabetes/obesity/asthma disproportionately? a. If so, what is/are the major arsenic contributor(s) to daily dose of arsenic?	• Gardenroots Data • USEPA’s EJSCREEN • National Water Quality Monitoring Counci • U.S. Geological Survey • ADHS Environmental Health Public Tracking • Behavioral Risk Factor Surveillance • National Health and Nutrition Examination Study • USDA ERS
6. Can we assign an index value (Bergstrand et al., 2015) ?	All datasets in Tables 1, 2
7. Once we combine the vulnerability and resiliencies, can we rate and compare communities?	N/A
8. How can we leverage the resiliencies to address the vulnerabilities?	All datasets in Table 2
9. When considering ecosystem functions, what function(s) are in deficit/not working? Which functions are working? a. Provisioning b. Regulating c. Cultural d. Supporting	All datasets in Tables 1, 2
10. When considering sustainability practices, what needs to occur: a. Economically—new job opportunities? b. Socially c. Environmentally	All datasets in Tables 1, 2
11. How can we successfully communicate with these communities at the local community and government level?	• American Community Survey

## Data Availability

The original contributions presented in the study are included in the article, further inquiries can be directed to the corresponding author/s.
